# Food Insecurity Screening and Referral Practices of Pediatric Clinicians in Metropolitan Washington, DC

**DOI:** 10.3390/children11091147

**Published:** 2024-09-23

**Authors:** Kofi Essel, Michael Burke, Laura Fischer, Mark Weissman, William Dietz

**Affiliations:** 1 School of Medicine and Health Sciences, George Washington University, Washington, DC 20052, USA; 2 Department of General & Community Pediatrics, Children’s National Hospital, Washington, DC 20010, USA; 3 Elevance Health, Indianapolis, IN 46204, USA; 4 Nutrition Consortium, Arnold School of Public Health, University of South Carolina, Columbia, SC 29208, USA; 5 The Sumner M. Redstone Global Center for Prevention & Wellness, Milken Institute School of Public Health, George Washington University, Washington, DC 20052, USA

**Keywords:** food insecurity, pediatrician, screening, primary care, social drivers of health

## Abstract

Background/Objectives: In 2022, 17.3% of US households with children experienced food insecurity (FI). The objective of this study was to examine pediatric clinicians’ FI screening and management immediately following the release of the American Academy of Pediatrics (AAP) 2015 FI Policy Statement. Methods: Data were collected in 2016 from 85 primary care pediatric clinicians via an online survey of clinicians in the Washington, DC metropolitan area. Descriptive statistics were calculated using univariate/bivariate analyses. Fisher’s exact test and Chi-square tests were used to explore the association between FI screening, health insurance, and clinician demographics. Results: Sixty-six percent of clinicians indicated that they infrequently screened for FI. Only 13% of clinicians used a standardized FI screening tool. Forty-five percent of clinicians screened for FI only when they perceived an acute concern. About 70% of them screened for FI when a patient presented with poor weight gain or was underweight. Conclusions: Immediately after the release of the AAP Policy Statement, it was found that few pediatric clinicians appropriately and frequently screened for FI in our regional sample. Our data emphasize the common misconceptions held by clinicians around FI and the necessity to incorporate training that underscores the invisibility of FI along with effective techniques to screen and intervene.

## 1. Introduction

Food insecurity (FI) describes “the limited or uncertain availability of nutritionally adequate and safe foods, or limited, or uncertain ability to acquire acceptable foods in socially acceptable ways” [1]. In 2022, 17.3% of households with children experienced FI compared to 12.8% of households without children [2]. Higher rates of FI compared to the general population are found in households experiencing poverty and near poverty, headed by a single caretaker, or having a parent with disabilities or maternal depression, and in families living in urban or rural communities or headed by Black, Indigenous, and Hispanic persons who are often directly connected to historical trauma, racism, and systemic injustices [2,3,4]. Identifying children in households experiencing FI is essential because of the adverse effects on their general health, iron deficiency, behavioral and emotional problems, higher emergency department utilization and hospitalization rates, and lower math and reading gains [5,6,7].

In 2024, a draft recommendation from the United States Preventive Services Task Force was released, which found insufficient evidence that screening to address FI within primary care healthcare settings improves overall health outcomes. Although insufficient evidence linked improved food security to health outcomes, the report acknowledged the “strong logic to support screening for and assistance ameliorating food insecurity, both because food is a basic human need…and because knowing that a patient is food insecure has important implications of the delivery of healthcare”. Pediatric clinicians are uniquely suited to address FI in the clinical setting [8,9,10]. In December 2015, the American Academy of Pediatrics (AAP) released a policy statement encouraging clinicians to use validated tools to screen patients and intervene to address FI [11]. The policy statement also provided resources and encouraged clinicians to advocate at the systems level to support households experiencing FI in their communities. However, few studies have examined clinician awareness and practices prior to and following the release of the policy statement [12,13]. Prior to the policy statement, one study reported that only 8% of pediatric clinicians considered themselves “very knowledgeable” about FI, and only 13% reported that they consistently asked about the sufficiency of food in their families’ homes [12,13].

General pediatric clinicians can play a critical role in identifying and addressing FI in the clinical setting. The goal of this pilot study was to generate estimates of pediatric clinician practices related to FI to inform a future large-scale study and educational intervention. This pilot study had three main aims with respect to the assessment of FI: (1) To examine the frequency and type of clinician screening; (2) To examine factors associated with clinician screening; (3) To explore the resources provided to families by clinicians to address FI. 

## 2. Materials and Methods

In 2016, we surveyed a sample of general pediatric clinicians immediately following the national release of the 2015 AAP FI Policy Statement in Washington, DC, and the surrounding metropolitan area [11]. Data were collected via an online survey that was sent to general pediatric clinicians in Washington, DC and the surrounding metropolitan area. We used a local community-based pediatric network, the Children’s National Health Network (CNHN). The CNHN reaches over 1500 pediatric clinicians in the Mid-Atlantic region and is one of the nation’s largest dedicated pediatric clinician networks. We sent the online survey to a subgroup of 380 pediatric clinicians in Washington, DC and the surrounding metropolitan area. To be included in the study, practitioners must have been the following: (1) An outpatient primary care pediatric clinician (i.e., general pediatrician, nurse practitioner, family medicine, or physician assistant) currently in practice; (2) Practicing at least 1-day per week; (3) Practicing in Washington, DC and the surrounding metropolitan areas in Maryland and Virginia with a valid email address; (4) Currently enrolled within the CNHN registry.

An online survey tool was designed to assess factors influencing primary care pediatric clinician’s knowledge, attitudes, and behaviors associated with screening for and addressing FI in their outpatient clinical setting. In addition, pilot-tested questions were obtained and used with permission from a clinician survey tool designed and implemented in 2007 [12]. Our survey tool was completed in 2014 and reviewed and tested by a total of 25 pediatricians, anti-hunger advocates, and biostatisticians to assess face validity. Study data were collected and managed using Research Electronic Data Capture (REDCap) tools hosted at Children’s National Hospital [14]. REDCap is a secure, web-based application designed to support data capture for research studies. The survey tool was distributed through a listserv and made accessible for 6-weeks from August to September 2016. Four reminder emails were sent during this period. Participants were also allowed to enter a raffle for two USD 50 gift cards used as incentives for successful completion of the survey. Children’s National Hospital Institutional Review Board approved the protocol and survey.

### 2.1. Variables of Interest

Sociodemographic factors included clinician credentials (e.g., general pediatrician, family medicine, nurse practitioner, physician assistant), clinician demographic characteristics (e.g., age, gender, race), and practice characteristics (e.g., years in practice, type of practice, insurance type). Pediatric clinicians were also asked general questions about their knowledge of FI using a 4-point Likert-item (no knowledge, somewhat/moderately/very knowledgeable). 

Clinicians were asked questions about practices related to FI, including the following: (1) Frequency of screening using a 5-point Likert scale (always, often, sometimes, rarely, or never); (2) Barriers to screening (i.e., time constraints and unknown resources); (3) Method of screening (i.e., verbal and written) using pre-identified choices with multi-checkbox response options. Use of a validated screening tool by clinicians was determined using a dichotomous response. Response options regarding factors that prompted clinicians to ask about FI were based on previously piloted questions using a 5-point Likert scale. Clinicians were also asked about referral behaviors after identifying families in households experiencing FI using a list of general pre-identified resources with a 5-point Likert scale.

### 2.2. Statistical Analysis

Analyses were conducted using SAS 9.3 (SAS Institute, Cary, NC, USA). We used descriptive statistics to examine the sociodemographic characteristics of the clinicians and the percentage of patients on public and private insurance, the number of days per week that clinicians saw patients, methods of clinician screening, location of screening, use of screening tools, FI prompts to screening, and referral practices. We tested the association between the clinician demographic and practice characteristics and clinician screening frequency using Fisher’s exact and Chi-square tests. All tests were two-tailed and used a significance level of 0.05. The number of cases in each analysis varied slightly because of missing values for specific questions.

## 3. Results

A total of 380 clinicians from the CNHN were invited to enroll in an online survey, and 114 responses were obtained. Twenty-nine responses were excluded because of missing data, because the respondent opted out of the survey before completion, or because the clinician was located outside of the study region. A total of eighty-five clinicians submitted complete data on all key variables, a response rate of 30%. Overall, the majority of respondents were pediatricians (86%). Clinicians mainly practiced in clinics in the District of Columbia (53%) or Maryland (29%) (Table 1).

The majority of clinicians were women (81%). In addition, 54% of respondents were White, 28% were Black, and 18% were considered “other” race. Most clinicians saw a larger proportion of patients who received public (>60%) rather than private insurance (<20%). Respondents saw patients on average 3 days per week within their medical practice and had been practicing on average >12 years (55%).

Fifty-two percent of clinicians indicated that they were “moderately” to “very knowledgeable” about FI. However, most clinicians (66%) indicated that they screened infrequently (sometimes, rarely, or never) for FI in their general pediatric clinical setting (Table 2). 

**Figure 1:**
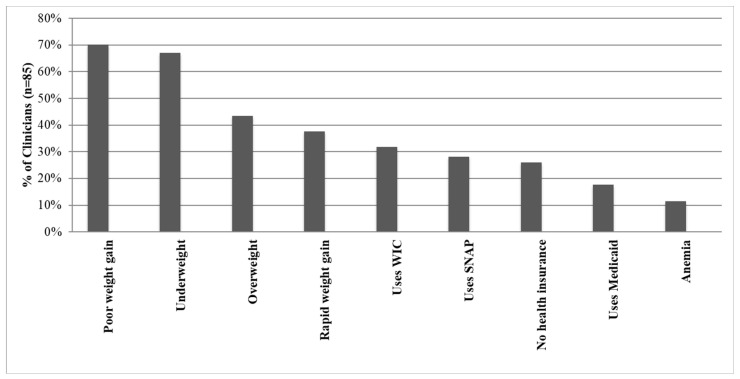
Indicators that Prompt Pediatric Clinicians in the Washington, DC Metropolitan Area to Ask about Food Insecurity.

The clinicians who regularly screened for FI described using a variety of techniques, including the majority (81%) verbally asking their families about FI; about 4% requested families to complete a written screening form (family completes independent of clinician); 3% involved their medical staff team; and 12% incorporated FI screening questions into their electronic medical records. Two-thirds of clinicians asked about FI in pediatric well child exams, and 8% asked during sick visits. Almost half (45%) of clinicians asked about FI only when they perceived an acute concern. Clinicians who screened for FI rarely used standardized screening tools (13%) and asked about FI based on specific findings such as anthropometry, insurance status, anemia, or use of federal nutritional programs. Respondents indicated that being overweight (44%) or having rapid weight gain (38%) was less of a prompt compared to being underweight (67%) or having poor weight gain (70%) (Figure 1).

**Figure 2:**
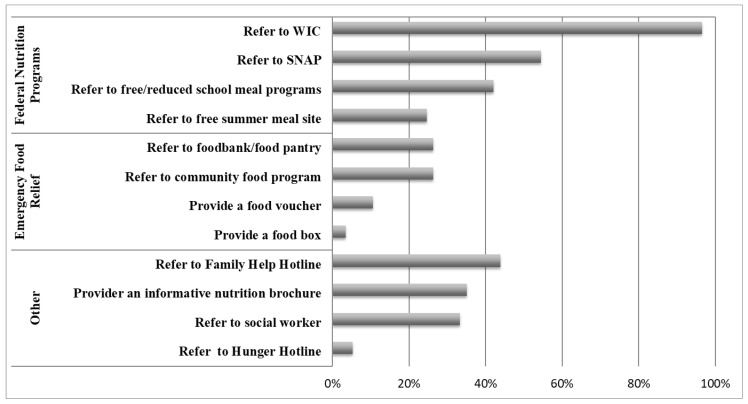
Referral Practices of Pediatric Clinicians in the Washington, DC Metropolitan Area for Households Experiencing Food Insecurity.

In addition, patients receiving The Special Supplemental Nutrition Program for Women, Infants, and Children (WIC) (31.8%) or the Supplemental Nutrition Assistance Program (SNAP, formerly known as the Food Stamp Program) (28%) were also less likely to receive FI screening compared to those not receiving either program (Figure 2).

Clinicians indicated that the most common resources recommended for families in households experiencing FI were federal nutritional programs, primarily SNAP (54%) and WIC (97%). Emergency food resources, such as food pantries and food banks (26%), were recommended less frequently. 

African-American clinicians were more likely to screen for FI compared to White or other races (*p* = 0.002, Table 3).

Similarly, individuals who saw a larger share of patients on Medicaid (*p* = 0.016) and a smaller share of privately insured patients (*p* = 0.033) were more likely to screen for FI. Other factors, such as years of practice, age, gender, and number of days practiced, were not significantly associated with screening.

## 4. Discussion

This cross-sectional pilot study describes the FI screening and referral practices of a mostly urban sample of general pediatric clinicians serving a majority under-resourced patient population in Washington, DC, and the surrounding metropolitan area. Our results suggest that immediately after the release of the 2015 AAP FI Policy Statement, most clinicians described being knowledgeable about FI. Despite this assertion, clinicians were not frequently or appropriately screening for FI and used a limited range of resources to intervene with their families. This is useful information for understanding the context in which the policy statement was released and the areas for potential improvement in policies and recommendations.

The well child examination offers pediatric clinicians more time to conduct a comprehensive assessment and examination of child and family social, physical, and mental concerns [15]. We observed that the majority of clinicians who screened for FI did so during these visits, which is consistent with AAP recommendations. However, we found that many clinicians infrequently screened for FI in their practice setting. Many practices may have limited capacity and lack the use of efficient methods to screen for FI, such as with electronic tablets, integration into electronic medical records, and working with staff. To maximize screening during the well child examination, clinicians could integrate social drivers of health screeners into electronic medical records to better measure, identify, refer, and follow up with families that are households experiencing FI. Strategies that rely on office staff before the actual health visit (e.g., screening during triage, in the waiting room, or an online portal) could improve the detection of FI [16].

We observed that many clinicians screened only when prompted by a concern. The most common concerns were changes in weight status, such as underweight or poor weight gain. Although these findings may assist clinicians in identifying some households experiencing FI, anthropometric and laboratory findings do not reliably identify FI [17,18]. We found that clinicians were more likely to ask about FI when a patient was underweight or experiencing poor weight gain than when the patient was overweight. The association with underweight status and FI is mixed because FI does not always lead to undernutrition that results in poor weight gain and growth [19,20]. In addition, the association of childhood obesity with FI is also mixed; studies indicate no association, a positive association, or a negative association [18,21]. However, theoretically, it is understood that factors that are associated with FI (i.e., low socioeconomic status and health disparities) may contribute to a family’s excessive caloric intake, decreased physical activity, increased stress levels, and weight gain [22,23]. Because excessive weight gain does not trigger the same nutritional concerns as poor weight gain, it is understandable why clinicians may fail to identify a significant number of patients and families experiencing FI. Ultimately, these external findings and implicit/explicit biases will underestimate and misidentify households experiencing FI in the clinical setting and should not be relied upon as a sole source of information to drive screening practices. 

FI is pervasive yet so infrequently recognized in clinical settings that it can be considered invisible to practitioners. In addition to the lack of clinical awareness, parents also try to hide their lived experiences of FI to protect their children from this stress. Parents often buffer the effects of FI on their young children by skipping meals so their children will not go hungry [2,3,24]. Although parental actions to protect their children may limit the harmful effects of food shortages, these households may not be able to avoid the increased psychosocial stressors associated with FI [25]. The invisibility makes it challenging for clinicians to identify FI in the clinical setting using traditional examination tools and methods [17,18]. The ideal tools to identify and manage FI for busy clinicians must be easily and universally administered, specific and sensitive, and provide the resources necessary for effective referrals [14,15,26]. 

We found that fewer than 25% of clinicians used standardized screeners to detect FI. Therefore, it is likely that the majority of clinicians were using unreliable tools to screen for FI, thereby potentially missing a large and vulnerable population. Many tools have been developed and discussed in the literature to screen for FI in clinical settings [2,17,27]. The AAP has supported the 2-question Hunger Vital Sign^©^ as the most efficient tool due to its ease of use and high sensitivity and specificity [11,17]. In addition, this tool serves as one of the most frequently used clinical tools to identify risk for FI. The Hunger Vital Sign^©^ serves as a simple validated tool to integrate into the clinical practice setting. These questions are “[w]ithin the past 12 months we worried whether our food would run out before we got money to buy more”, and “[w]ithin the past 12 months the food we bought just didn’t last and we didn’t have money to get more” [11,17]. In addition to using inadequate and non-standardized screening practices, clinicians also described most consistently verbally asking about families’ food security as their preferred methodology as compared to a written screener. Although this strategy is practical and efficient, because of the increased sensitivity, shame, and guilt around social drivers of health screening, this may lead to under-reporting because the data suggest a higher prevalence of positive screening has been observed when families respond to a written/electronic form vs. verbal screening practices [28,29].

Screening practices for social drivers of health, such as FI, are often limited in the clinical setting because of insufficient time, inadequate training, lack of resources, and sensitivity of questions [26]. Questions related to FI may even be perceived as intrusive, with some families even voicing concern about child protective services being summoned if they share these hardships in clinical settings [29]. Many physicians, including the majority of our sample, reported coming from middle to upper income backgrounds [30]. Income disparity may contribute to a decreased desire to probe into a patient’s lived experience of health disparities and a reluctance to ask sensitive questions about a family’s social history [31]. In addition, this socioeconomic discordance between clinicians and patients may limit clinicians’ ability to effectively engage patients and make appropriate and practical recommendations that adequately address social concerns. 

We observed that clinicians were more likely to screen for FI in populations that have a higher Medicaid participation rate. Clinicians who care for a larger number of patients who receive public insurance may be more sensitive to social concerns and, thus, more vigilant about screening for FI. Furthermore, African-American clinicians appeared more likely to ask about FI in our sample than their colleagues. Because metropolitan DC has a large African-American population, racial concordance may lead to an increased level of comfort in addressing FI, but the evidence is less defined in the pediatric population [32,33]. African-Americans and other clinicians of color are more likely to work in marginalized areas, and we found a higher frequency of screening among clinicians who worked in more under-resourced, higher publicly insured populations [34,35]. 

Fewer clinicians asked about FI if the family was already connected to federal nutrition programs such as WIC and SNAP. Clinicians may assume that these patients are already “connected” and may no longer be experiencing FI. However, this potential gap in referrals to federal nutrition programs highlights an important concern for households experiencing FI. Patients who receive WIC or SNAP represent a different group than those who are also in poverty but are not receiving WIC or SNAP. Generally, patients who receive WIC or SNAP may represent a more vulnerable group than those not receiving these services [2,3]. In addition, benefits from nutrition programs such as SNAP prior to the October 2021 historic pandemic increase have been inadequate for most families needing the support; 60% and 80% of SNAP dollars are typically spent by day 7 and 14, respectively, within the month’s allotment [36]. These patients and families may need even more access to resources than they currently receive in many pediatric primary care practices. Our study showed that clinicians importantly relied heavily on social safety net programs, primarily WIC and SNAP, as a first line of defense for battling FI. The 40% difference in referral trends for WIC versus SNAP may be due to the combined application for SNAP/Medicaid/Temporary Assistance for Needy Families (TANF) applications that were available in the DC Metropolitan area. Therefore, patients who signed up with Medicaid could automatically sign up with SNAP, and clinicians may not see the same gaps in enrollment as they see with the WIC program [37]. In addition, because WIC is only offered to children between the ages of 0 and 5 years, there are limitations in referral trends due to lack of availability. Lastly, one-quarter of clinicians referred patients to emergency food resources, such as food banks or pantries. Emergency resources are an essential component of food security for many families with FI because many families may be uncomfortable or unable to access federal nutrition programs, and 30% of families with FI are financially ineligible for federal nutrition programs such as SNAP, WIC, and School Meals [2]. However, emergency/charitable resources are limited in their ability or capacity to manage FI longitudinally for families. Clinicians may be less familiar with local community emergency resources for their patients and families, thereby limiting their use. Training clinicians to recognize and partner with local anti-hunger organizations and charitable food resources is essential to the clinical management of FI. Lastly, the concept of “Food as Medicine”, strategies that align with healthcare and use food to improve health, has gained great momentum in recent years. These novel strategies that incorporate other interventions, such as medically tailored meals, medically tailored groceries, and produce prescriptions, can also be considered a meaningful strategy to optimize the management of children and families experiencing food insecurity often complicated by other co-occurring diet-related disease risks [38,39,40]. These strategies often require effective food and nutrition insecurity screening at baseline, so ensuring evidence-based screening and intervention approaches in health systems is a key component to scaling Food as Medicine programs across the country. This paper hopes to shed more light on previous screening and intervention practices and the importance of continuing to improve future healthcare infrastructure.

There are several limitations to our study. Consistent with prior studies surveying physicians, we encountered a low response rate [41]. In addition, the CNHN listserv frequently directs emails to office staff who were requested to disseminate surveys to clinicians, which may have been a rate-limiting factor for clinician responses. The limited response rate increases the potential for response bias. Clinicians who were more interested in this topic may have been more likely to respond [42]. As a result, our findings may overestimate engagement with FI because non-responders may be less engaged with issues covered in our study. Because the 2015 AAP FI Policy Statement was released before the dissemination of our survey, screening may have been increased due to the increased attention to FI. Lastly, it is important to remember that these data were collected prior to the COVID-19 pandemic when food insecurity was amplified in the public spotlight. However, the authors agree that the momentum around this topic continues to make it relevant for current discussion.

Despite these drawbacks, and while the results are several years old, our study provides a unique insight as a historical context and fills a knowledge gap by providing observational data on the experiences of busy and hard-to-reach pediatric clinicians around FI. In addition, another strength was that we acquired a pretested survey instrument and combined previously used questions to assess our population. Lastly, our topic is very timely and remains an active area of research that may inform policy discussions on moving forward with effective ways to screen and intervene to train future providers in the clinical setting and scale effective food as medicine interventions.

## 5. Conclusions

Our results identified gaps in screening and referral practices of pediatric clinicians’ status post-release of the 2015 AAP FI policy statement. Implementation of these recommendations, including universal screening for FI, will continue to require improved training in social drivers of health, strategies to incorporate these practices into busy primary care practices, and team and community-centered approaches. Systemic strategies that encourage diversification of the clinical workforce may provide increased sensitivity in screening within clinical practices. In addition, clinicians will need resources to help connect families to federal nutrition programs, emergency food resources, and financial support to sustainably assist families through times of food hardship. Finally, because FI is often experienced in combination with income hardship and other social drivers of health, the development and validation of comprehensive psychosocial screeners that can be integrated into busy clinical practices is essential.

## Figures and Tables

**Table 1:** Descriptive statistics of pediatric clinicians and practices in the Washington, DC metropolitan area (n = 85).

Variable	Percentage (n)
Age (years)	
25–34	22.4 (19)
35–44	29.4 (25)
45–54	22.4 (19)
>55	25.8 (22)
Gender	
Female	81.2 (69)
Male	18.8 (16)
Provider Type	
Pediatrician	85.9 (73)
Family Medicine (MD)	2.4 (2)
Nurse Practitioner	10.6 (9)
Physician Assistant	1.2 (1)
Race or ethnicity	
White, non-Hispanic	54.1 (46)
Black, non-Hispanic	27.1 (23)
Other race or ethnicity	18.8 (16)
Years of Clinical Practice	
0–11	43.5 (37)
12–23	36.5 (31)
24 or more	20 (17)
Location of Pediatric Practice	
District of Columbia	53 (45)
Maryland	29.4 (25)
Virginia	12.9 (11)
More than 1 State	4.7 (4)
Practice Setting ^1^	
Urban	70.6 (60)
Suburban	33 (28)
Rural	1.2 (1)
Percentage of patients using Medicaid ^2^	
0–40%	35.3 (30)
41–100%	64.7 (55)
Percentage of patients using private insurance ^2^	
0–40%	62.4 (53)
41–100%	37.6 (32)
Number of days clinician sees patients per week	
≤2 days	23.5 (20)
3 or more days	76.5 (65)

^1^ Respondents could select multiple responses. Percentages represent percent of total responses and (n) indicates number of respondents who select response. ^2^ Pediatric clinician’s perceived percentage of patients using Medicaid or private health insurance.

**Table 2:** Food insecurity screening practices and prompts to ask about food insecurity among pediatric clinicians in the Washington, DC metropolitan area.

Question	Percentage (n)
Screen for food insecurity (n = 85)	
Always or often	34.1 (29)
Sometimes, rarely, or never	65.9 (56)
Method of screening for food insecurity (n = 84) ^1^	
Verbal by pediatric clinician	85.7 (72)
Other by other medical staff	3.6 (3)
Patient completes written form	3.6 (3)
Clinician uses Electronic Medical Record	13.1 (11)
When do clinicians screen for food insecurity (n = 84) ^1^	
All Visits	0 (0)
Well Child Visits	66.7 (56)
Sick Visits	8.3 (7)
Only when there is a concern	45.2 (38)
Use of Standardized Screening tool (n = 72) ^2^	
Yes	12.5 (9)
No	87.5 (63)
Ask about food insecurity if patient (n = 85) ^1,3^	
Has poor weight gain	70.1 (60)
Is underweight	67.1 (57)
Is overweight	43.5 (37)
Has rapid weight gain	37.6 (32)
Uses WIC	31.8 (27)
Uses SNAP	28.2 (24)
Has no health insurance	25.9 (22)
Uses Medicaid	17.6 (15)
Is anemic	11.5 (39)
Referral practices if patient in food insecure household (n = 57) ^1,3^	
Refer to WIC	96.5 (55)
Refer to SNAP	54.4 (31)
Refer to free/reduced school meal programs	42.1 (24)
Refer to free summer meal site	24.6 (14)
Refer to foodbank/food pantry	26.3 (15)
Refer to community food program	26.3 (15)
Provide a food voucher	10.5 (6)
Provide a food box	3.5 (2)
Refer to Hunger Hotline	5.3 (3)
Refer to Family Help Hotline	43.9 (25)
Refer to social worker	33.3 (19)
Provide an informative nutrition brochure	35.1 (20)

^1^ Respondents could select multiple responses. Percentages represent percent of total responses, and (n) indicates number of respondents who selected response. ^2^ Number of respondents varies as result of partial opt-out or non-response. ^3^ Percentage (n) represent pediatric clinicians who affirmed either “Always” or “Often”.

**Table 3:** Association between pediatric clinician screening for food insecurity and demographic and clinical factors in the Washington, DC metropolitan area.

Clinician Demographic Variable	Food Insecurity Screening Response		*p*-Value
	Always or Often % (n)	Sometimes, Rarely, or Never % (n)	
Age (years) ^1^			0.975
25–34	8.2 (7)	14.1 (12)	
35–44	9.4 (8)	20.0 (17)	
45–54	7.1 (6)	15.3 (13)	
>55	9.4 (8)	16.5 (14)	
Gender ^2^			0.775
Male	7.1 (6)	11.8 (10)	
Female	27.1 (23)	54.1 (46)	
Race or ethnicity ^1^			0.002
White, non-Hispanic	9.4 (8)	44.7 (38)	
Black, non-Hispanic	14.1 (12)	12.9 (11)	
Other race or ethnicity	10.6 (9)	8.2 (7)	
Percentage of patients using Medicaid ^2,3^			0.016
0–40%	5.9 (5)	29.4 (25)	
41–100%	28.2 (24)	36.5 (31)	
Percentage of patients using private insurance ^2,3^			0.033
0–40%	27.1 (23)	35.3 (30)	
41–100%	7.1 (6)	30.6 (26)	
Number of days clinician sees patients per week ^2,3^			0.423
≤2 days	5.9 (5)	17.6 (15)	
3 or more days	28.2 (24)	48.2 (41)	

^1^ Chi-square test was used to calculate *p*-values. ^2^ Fishers exact test was used to calculate *p*-values. ^3^ Pediatric clinician’s perceived percentage of patients using Medicaid or private health insurance.

## Data Availability

The original contributions presented in the study are included in the article; further inquiries can be directed to the corresponding author.
